# Effects of maternal morphine consumption on odontogenesis in rats: A histomorphometric study

**DOI:** 10.34172/joddd.025.40947

**Published:** 2025-03-31

**Authors:** Mahsa Kalantari, Yasamin Shahsavani, Massood Ezzatabadipour, Hoora Shoja Alsadati, Maryam Eslami, Sara Amanpour

**Affiliations:** ^1^Oral and Dental Diseases Research Center, Department of Oral and Maxillofacial Pathology, Kerman Dental School, Kerman University of Medical Sciences, Kerman, Iran; ^2^Department of Anatomical Sciences, School of Medicine, Kerman University of Medical Sciences, Kerman, Iran

**Keywords:** Morphine, Odontogenesis, Opium dependence, Rats

## Abstract

**Background.:**

Extensive research has established the adverse impact of morphine sulfate addiction on the central nervous system. Additionally, studies have shown that the consumption of morphine during pregnancy can impair normal fetal development. This study examined the influence of morphine sulfate on tooth development in rats.

**Methods.:**

Thirty female Wistar rats were randomly assigned to two groups. The experimental group was given morphine sulfate at a final dose of 0.4 mg/mL, while the control group received only water. Dependence on morphine was verified through the use of naloxone. We examined the effect of morphine sulfate on the development of maxillary first molars in rat embryos on day 19 of gestation, as well as on days 1, 4, 7, and 10 after birth. Two or three rats were separated from each mother and anesthetized using ketamine at 2 mg/kg. Thin sections were prepared from paraffin blocks of the tissue and stained with hematoxylin and eosin. Statistical analyses were conducted with SPSS 20 using ANOVA and post hoc Tukey tests (*P*<0.05).

**Results.:**

Research findings indicated that, on the first day after birth, the enamel organ had a significantly greater thickness in the control group (*P*=0.001). Additionally, in the 19-day-old fetus and one-day-old baby groups, the control group had a significantly higher dental papilla thickness than the experimental group (*P*=0.001). Furthermore, the maximum buccolingual width of tooth buds in the control group was significantly greater than that of the experimental group (*P*=0.001). Lastly, the enamel and dentin were significantly thicker in the control group than in the experimental group in the 1-, 4-, 7-, and 10-day-old infant groups (*P*=0.001).

**Conclusion.:**

These results suggest that morphine sulfate interfered with the development of the tooth bud and reduced the secretion of enamel and dentin matrix.

## Introduction

 Drug addiction is a physical response to the repeated use of addictive substances. While it may provide temporary relief, relaxation, or excitement, the body will crave the substance once its effects wear off, leading to dependence.^1,2^ Shockingly, according to United Nations reports, over 300 million people worldwide are addicted to drugs. The opioid epidemic is currently a significant public health crisis, with opioid overdose deaths rising from 69 000 to 118 000 in just one year, according to the World Health Organization.^3^ Unfortunately, opioid use during pregnancy has also increased over the past decade, leading to infant abandonment syndrome. Research shows that women typically begin drug use during their reproductive years, with an average age of 27.5 ± 10.6 years.^4^ Morphine, a commonly abused opioid, can pass through the placental barrier and affect fetal tissues, including fatty tissues. In addition, morphine use during pregnancy can lead to decreased placenta weight, as well as decreased fetal weight and length.^5,6^

 The formation and development of teeth is a process that is similar across most mammals. This process, known as odontogenesis, involves interactions between the dental lamina from the oral epithelium and the ectomesenchyme from the neural crest.^7^ Studies on rodent teeth, specifically rats, have contributed the most knowledge about odontogenesis.^8^ However, certain factors like trauma, bacterial and viral infections, maternal malnutrition, drug use, smoking, and alcohol can impact the development of tooth buds. Research has shown that alcohol,^9^ nicotine,^10^ and fluoxetine^11^ can have adverse effects on dental development in rats. In a study on rats, Sant’Anna and de Oliveira Tosello^9^ showed that alcohol consumption during pregnancy can result in smaller tooth buds and less enamel matrix formation in infants. Chowdhury and Bromage^10^ also stated that nicotine can delay tooth development and disrupt cell maturation in the tooth bud. Furthermore, Avsar et al^12^ found that children between the ages of 4–6, who were exposed to cigarette smoke at home, had significantly lower dental development than the control group.

 Based on the materials we have reviewed and our internet search, it appears that the impact of morphine on dental development has not been studied in humans. Most of the information on odontogenesis comes from research on rodents, particularly rats. Our study aimed to explore the effects of morphine use during pregnancy on dental development in rat fetuses and infants, comparing our findings with those of a control group.

## Methods

###  Animals

 In this study involving animals, 30 healthy female Wistar rats aged 6–8 weeks were provided with appropriate conditions such as a temperature range of 21–23 ºC, 12 hours of light and 12 hours of darkness, and sufficient food and water. The rats were obtained from the animal house of Afzalipur Medical School and randomly divided into two groups. The experimental group consisted of 15 rats who became dependent on morphine, while the control group comprised 15 rats not dependent on morphine. In order to induce morphine dependence in female rats, the experimental group was given access to drinking water containing morphine. To reduce the stress of repeated injections during pregnancy, morphine was administered orally and added to the drinking water. To make the taste more palatable, sucrose was added at 2 g/L. The concentration of morphine varied over time, with 0.1 mg/mL for the first two days, 0.2 mg/mL for the second two days, and 0.3 mg/mL for the third two days. From the 7th to the 15th day, the concentration was 4 mg/mL. This dose was continued until the end of pregnancy and breastfeeding. The control group was given plain drinking water. Both groups were given the same dry diet. The researchers examined the addiction of rats in the experimental group after 15 days of receiving morphine. They used intraperitoneal administration of naloxone, with a dosage of 2 mg/kg, to investigate withdrawal syndrome symptoms. After 30 minutes of injection, they monitored symptoms such as jumping, body stretching, drooping eyelids, curling up, shaking the head, excitability to touch, and particularly diarrhea. The rats that exhibited these symptoms were considered dependent. During the next step of the experiment, both the control and experimental groups of female rats were paired up with healthy male rats that were not addicted to morphine with the intention of mating. After 24 hours, the presence of vaginal plaque was used as evidence of successful mating, and the rats that displayed this were placed in separate cages. The date of the positive vaginal plaque was considered the first day of pregnancy, which lasts for 21 days in rats. If the vaginal plaque was not present, the non-pregnant female rats were separated from the males and returned to the cages of their respective groups after 24 hours.^4^ After confirming the pregnancy of both the experimental and control group rats, they were divided into 5 separate groups (10 groups in total). The first group consisted of rats whose embryos were removed on the 19th day of pregnancy. The second to fifth groups included rats whose newborns were killed on the 1st, 4th, 7th, and 10th days after birth. The study examined 80 samples (40 from the control group and 40 from the experimental group), with 2 to 3 fetuses or newborns selected from each female rat.^9-11^ To carry out surgery on pregnant rats, the initial group were given a deep anesthesia via an intraperitoneal injection of 600 mg/kg chloral hydrate on the 19th day of pregnancy. The embryos were then removed, and their separated heads were taken to the pathology department. The other four groups underwent the same procedures but on babies born on the 1st, 4th, 7th, and 10th days after birth, respectively.^9^

###  Histological processing

 Once the samples were transferred to the pathology department, they were fixed in a 10% formalin solution for 48 hours. Next, the samples were confirmed before being immersed in a 5% nitric acid solution for 72 hours to be decalcified. The next steps involved processing the samples, preparing paraffin blocks, and obtaining 3–5-µm coronal sections.^9-11^ Finally, the tissue samples were stained using hematoxylin-and-eosin staining.

###  Histomorphometry

 The stained slides, coded by a laboratory expert, were examined using a Nikon YS100 optical microscope from Japan equipped with an eyepiece micrometer. Each sample underwent a morphological examination of the maxillary right first molars. The thickness of the enamel organ, dental papilla, the greatest buccolingual thickness of the tooth bud, and the greatest thickness of enamel and dentin in the tip of the cusps were measured in micrometers. The pathologists, calibrated with each other, viewed the slides, and a third pathologist was consulted in cases of disagreement. All the samples were observed independently and separately without knowledge of their nature.

###  Statistical analysis

 The results were analyzed using SPSS 20. Since the results of the Kolmogorov-Smirnov test showed that the data had a normal distribution, the statistical tests using descriptive statistical methods were ANOVA and post hoc Tukey tests. A significant level of 0.05 was considered.

## Results

 The average number of babies per delivery in the control group rats (12.4 ± 0.7) was significantly higher than the experimental group (10.6 ± 0.8) (*P* = 0.001). However, the average weight gained by the mother rats in the control group during pregnancy (199.18 ± 10.19 g) showed no statistically significant difference from the rats in the experimental group (197.43 ± 9.48 g) (*P* = 0.707).

 The average weight of 1-day-old babies in the control group (6.41 ± 0.17 g) was significantly higher than the experimental group (5.35 ± 0.12 g) (*P* = 0.001). This difference was also significant in 4-, 7-, and 10-day-old babies (*P* = 0.001). In addition, the length of 1-day-old babies in the control group (68.2 ± 7.03 mm) was significantly higher than the experimental group (56.14 ± 5.13 mm) (*P* = 0.001), and a similar significant difference was reported in the studied groups (*P* = 0.001).

 This study examined 80 right maxillary first molars (including 5 groups of 8 mice from the experimental group and 5 groups of 8 from the control group). The cellular components of the enamel organ and dental papilla, their structural characteristics such as developmental stage, cell shape and type, blood vessels, and the amount of deposition of enamel and dentin matrix were morphologically examined in all the samples. As a result, no case of tooth agenesis was seen in the experimental or control group, and a continuous process of tooth development stages was seen in both groups.

###  19-Day-old embryos

**Figure 1:**
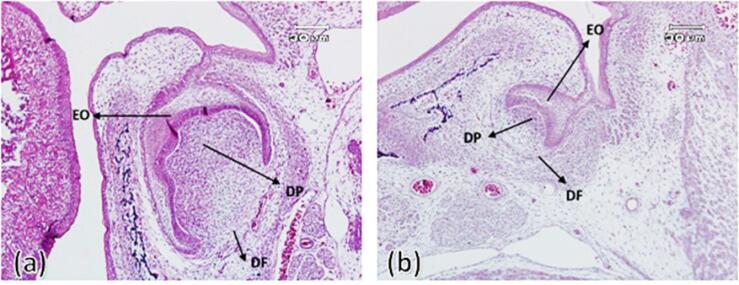
First maxillary molar tooth bud on the 19th embryonic day in the control group (a) and experimental group (b). Enamel organ (OE), dental papilla (DP), dental follicle (DF) ( × 100)

 In this group, the connection of the dental organ was observed in both experimental and control groups by the dental lamina appendage to the oral epithelium. Both groups exhibited a density of ectomesenchyme cells around the tooth bud (dental follicle) and under the concavity of the enamel organ (dental papilla). The enamel organ in both groups had clear boundaries and consisted of outer enamel epithelium, stellate reticulum, and inner enamel epithelium. Also, the enamel organ was separated from the underlying ectomesenchyme by an extended basement membrane and clear zone. Outer enamel epithelial cells were characterized as a continuous layer of short cylindrical cells with oval nuclei. The stellate reticulum was in the early stages of formation and was characterized by intercellular spaces and cells with diverse morphology (Figure 1).

###  The first day after birth

**Figure 2:**
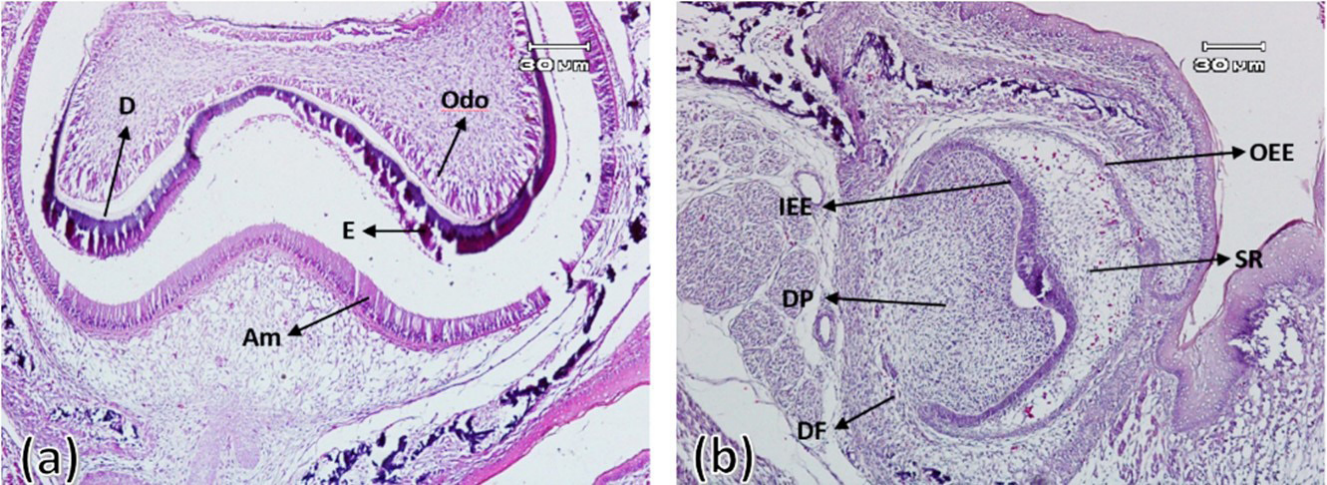
First maxillary molar tooth bud on the first day after birth in the control group (a) and experimental group (b). Outer enamel epithelium (OEE), stellate reticulum (SR), inner enamel epithelium (IEE), ameloblast (Am), enamel (E), and dentin (D) ( × 100)

 On this day, bone formation surrounding the tooth bud (alveolar bone formation) was observed, although much less bone was formed in the experimental group compared to the control group. The enamel organ in both groups had specific boundaries and consisted of external and internal enamel epithelium and stellate reticulum. Outer enamel epithelium cells were characterized as a layer of cylindrical cells surrounding the stellate reticulum (consisting of stellate cells with intercellular spaces). In the control group, numerous blood vessels that entered from the dental follicle towards the stellate reticulum were observed, which was less in the experimental group. In the control group, elongated cylindrical ameloblastic cells with nuclei arranged towards the intermediate layer and the differentiation of elongated cylindrical odontoblast cells with nuclei with reverse polarity were evident. In addition, the enamel and dentin matrix production had also started from the tip of the future dental cusps in all control group samples. In the experimental group, there was evidence of the differentiation of ameloblasts and odontoblasts and the beginning of dentin formation in only two samples. However, in the other six samples, there was no evidence of the differentiation of ameloblasts and odontoblasts and the formation of dental hard tissues (Figure 2).

###  Four, 7, and 10 days after birth

**Figure 3:**
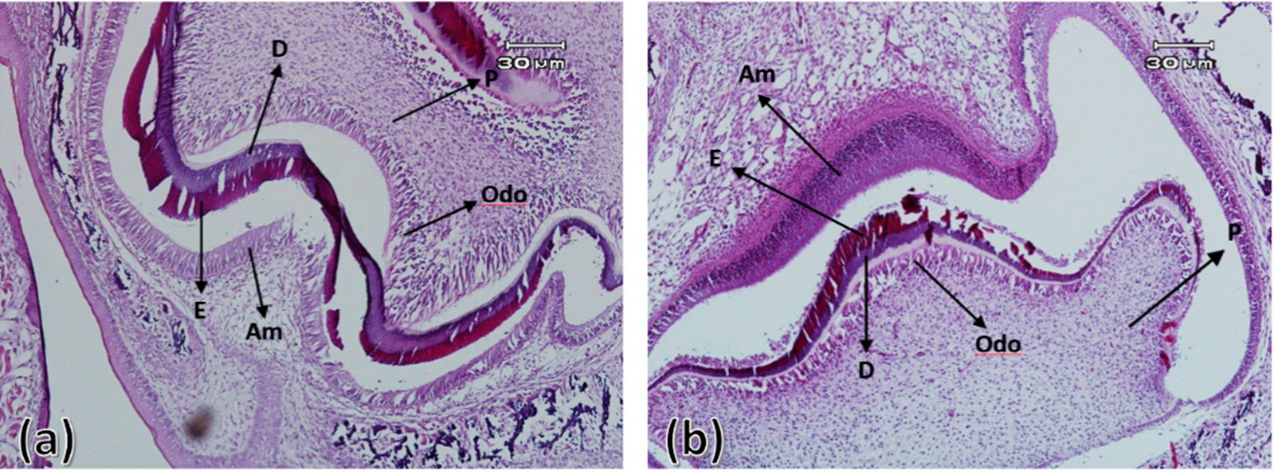
First maxillary molar tooth bud on the fourth day after birth in the control group (a) and experimental group (b). Ameloblasts (Am), odontoblasts (Odo), enamel (E), dentin (D), and pulp (P) ( × 100)

**Figure 4:**
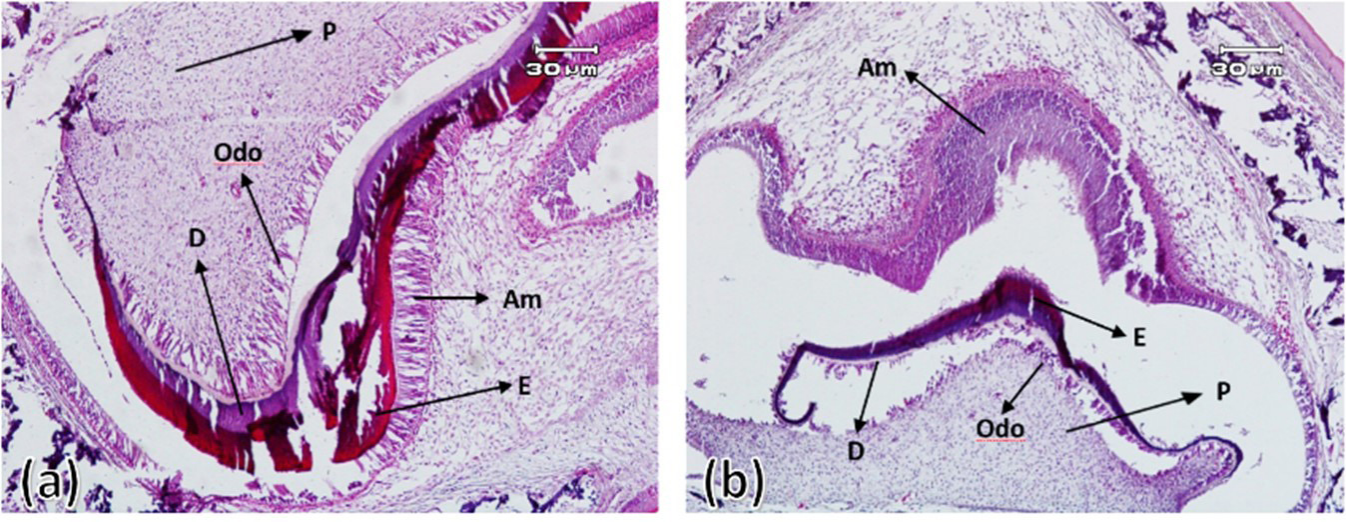
First maxillary molar tooth bud on the seventh day after birth in the control group (a) and experimental group (b). Ameloblasts (Am), odontoblasts (Odo), enamel (E), dentin (D), and pulp (P) ( × 100)

**Figure 5:**
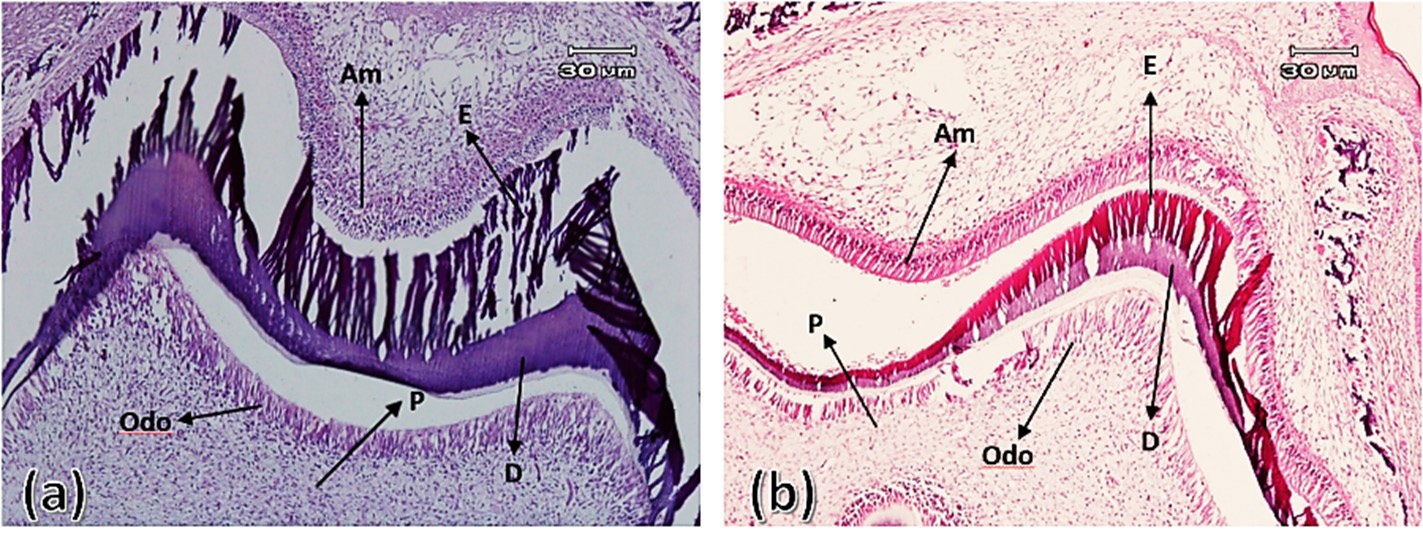
First maxillary molar tooth bud on the tenth day after birth in the control group (a) and experimental group (b). Ameloblasts (Am), odontoblasts (Odo), enamel (E), dentin (D), and pulp (P) ( × 100)

 On the fourth day after birth, the differentiation of ameloblasts, odontoblasts and the formation of enamel and dentin matrix were observed in all experimental group samples. In all samples of the control group, enamel and dentin production continued. On the 7th and 10th days after birth in the control group, the ameloblastic layer with elongated cylindrical cells was seen almost along the entire length of the crown of the tooth bud. The stellate reticulum had collapsed and was merging with the outer enamel epithelium, and a greater thickness of enamel and dentin had been built compared to postnatal day 4. In the experimental group, compared to the control group, less hard tissues were formed (Figures 3 to 5).

 In addition to examining the morphological characteristics, the thickness of the enamel organ, dental papilla, the maximum buccolingual thickness of the tooth bud, and the maximum thickness of enamel and dentin in the tip area of the cusps were measured in micrometers using an optical microscope. Table 1 presents the results of the different studied groups.

**Table 1:** The thickness of the enamel organ, dental papilla, the highest buccolingual thickness of the tooth bud, and the highest thickness of enamel and dentin in the area of the tips of the cusps in the different study groups

**Group**		**Variable**				
		**thickness of the enamel organ**	**Thickness of dental papilla**	**The greatest buccolingual thickness**	**Enamel thickness**	**Dentin thickness**
19-day old fetus	Control	5.34 ± 2.44	75.149 ± 67.9	25.194 ± 53.8	0	0
	Experimental	62.22 ± 66.3	25.43 ± 18.10	37.85 ± 89.23	0	0
1-day old fetus	Control	25.77 ± 94.7	37.185 ± 86.12	37.273 ± 43.12	75.9 ± 69.3	12.13 ± 15.4
	Experimental	25.33 ± 62.6	71 ± 53.13	127 ± 16.18	0	87.1 ± 56.3
4-day old fetus	Control	87.65 ± 64.9	149 ± 54.6	12.285 ± 31.7	25.23 ± 24.3	75.27 ± 45.3
	Experimental	37.65 ± 44.15	62.150 ± 63.8	25.195 ± 20.22	37.9 ± 26.2	50.11 ± 61.2
7-day old fetus	Control	25.51 ± 51.8	75.141 ± 12.7	87.292 ± 51.4	75.71 ± 77.4	50.80 ± 65.5
	Experimental	54 ± 54.3	62.143 ± 81.9	87.217 ± 34.18	75.14 ± 12.2	12.20 ± 58.2
10-day old fetus	Control	37 ± 41.6	62.141 ± 30.7	75.301 ± 01.19	25.83 ± 36.4	75.94 ± 26.4
	Experimental	45 ± 80.6	25.136 ± 68.8	75.233 ± 95.17	125.30 ± 90.2	50.39 ± 20.3

 The results of post hoc Tukey analysis comparing the thickness of the enamel organ between the control and experimental groups showed that the thickness of the enamel organ in the 1-day-old infants of the control group was significantly higher than the 1-day-old infants of the experimental group (*P* = 0.001).

 Comparing the thickness of the dental papilla between the control and experimental groups showed that only in 19-day-old fetuses and in 1-day-old babies the thickness of the dental papilla in the control group was significantly higher than in the experimental group (*P* = 0.001).

 In all studied groups, the maximum buccolingual thickness of the tooth bud in the control group was significantly higher than in the experimental group (*P* = 0.001 between all groups).

 The comparison of the maximum thickness of enamel and dentin between the samples of the control and experimental groups showed that in the groups of 1-, 4-, 7-, and 10-day-old control infants, the thickness of enamel and dentin was significantly higher than that of the experimental group infants (*P* = 0.001 between all groups).

## Discussion

 Numerous studies have assessed the impact of various drugs and medications used by pregnant mothers on the development of embryonic tooth buds in laboratory animals.^9-11,13-16^ However, an internet search showed that the effect of opioids on embryonic development in experimental animals during pregnancy has not been researched. The objective of this study was to gain a better understanding of how the consumption of morphine during pregnancy affects odontogenesis in rats.

 The present study showed that infants aged 1, 4, 7, and 10 days in the experimental groups had significantly lower enamel and dentin thickness than the control groups. Research has previously shown that different factors and diseases, such as hormonal, nutritional, systemic, genetic, and environmental factors, can interfere with amelogenesis. These factors impact the life cycle of ameloblasts and amelogenesis, causing various enamel defects.^17^ Sant’Anna et al^16^ reported that consuming ethanol can hinder the functioning of epidermal growth factor (EGF) receptors, affecting the differentiation of ameloblasts. This, in turn, can lead to a decline in the activity of ameloblasts and mineralized matrix production. According to Hernández Guerrero et al,^18^ newborn rats treated with ethanol exhibited smaller tooth buds compared to those not exposed to the substance. Moreover, ethanol intake during pregnancy decreased EGF expression in molar tooth follicles of infants, resulting in a reduced size of the tooth germ. Campos^19^ also noted cellular modifications in the tooth germ of rats during the early stages of bud caused by ethanol consumption. The most significant changes were observed in the local mineral epithelium (inter-enamel epithelium), which eventually differentiates into ameloblasts and begins producing enamel. The reduction in matrix production can be attributed to these changes. A study by Aycicek et al^20^ suggests that nicotine can adversely impact mineral metabolism by causing oxidative stress. Additionally, nicotine may lead to tissue breakdown and impaired crystallinity of hard tissue due to increased tissue oxygenation. Similarly, research by De Souza et al^15^ found that amoxicillin has the potential to inhibit ameloblasts’ differentiation, resulting in decreased enamel thickness. In a separate study, Abe et al^21^ discovered that administering macrolides during amelogenesis in rats’ incisor teeth can cause the degradation of ameloblasts in restricted areas, leading to hypomineralization in enamel. Finally, Westergaard^22^ reported structural changes in the ameloblast and matrix of tooth enamel due to tetracycline treatment. These findings suggest that the drug may have an inhibitory effect on ameloblasts’ maturity.

 This study revealed a correlation between variations or reduced thickness in the mineralized matrix and ameloblasts lacking autophagic-free constituents. Bronckers et al^23^ noted that while ameloblasts resist fluoride during the morphological phase, pre-ameloblasts are sensitive to average fluoride doses, which can alter cell structure and decrease protein synthesis. Any changes in enamel secretion by ameloblasts can impact the organic composition of the mineralized matrix or the mineral content. Amelogenins play a critical role in enamel secretion, forming small spherical structures (nanospheres) that eventually create a complex structure. This structure moderates the sediment and orientation of hydroxyapatite crystals. Additionally, other proteins like enamelin, growth factors, and proteases contribute to enamel formation through a complex molecular and cellular cascade of events.^24^ The study in question did not assess particular factors that could potentially be influenced by morphine. MMP20, an enzyme that assists in early mineralization processes and promotes the development of hydroxyapatite crystals by breaking down enamel protein, is believed to be integral to the demineralization process. Additionally, KLK4 plays a role in regulating crystal thickness during the final mineralization and maturation phase. As such, it is imperative to analyze both of these proteins when evaluating enamel’s mineralization and maturation stages. De Souza et al^15^ reported a significant difference in MMP-20 levels between mice that consumed amoxicillin and the control group during the secretion phase of their first molar at seven days old. To accurately analyze the final stages of enamel mineralization, which occur at the end of adolescence, it is important to determine the age of the studied rats. However, it is worth noting that examining inorganic enamel and mature enamel requires deceleration. This presents a challenge for immunohistochemistry research looking at enzymes involved in dental enamel mineralization^25^ because the enamel loses its organic matrix formation during the final stages of maturation due to its high mineral content when decalcified.

 According to our study, the buccolingual tooth germ’s maximum thickness was significantly lower in all experimental groups compared to the control groups. This finding is supported by previous studies from Sant’Anna and de Oliveira Tosello^9^ and Chowdhury and Bromage,^10^ who also reported similar results when investigating the effects of alcohol and nicotine consumption in rats during pregnancy. Other research has shown that maternal smoking can lead to smaller head sizes in newborns, while alcohol consumption can directly impact fetal growth and cause weight loss in babies.^26,27^ The rats born to morphine-dependent mothers in this study had a significantly lower weight and length compared to the control group. Finally, the thickness of the enamel organ in the one-day experimental group was significantly lower than the control group, but no significant difference was observed in the other groups.

 Recent research conducted by Chowdhury and Bromage^10^ and Avsar et al^12^ has highlighted the detrimental effects of nicotine consumption during pregnancy on dental germ development. The interaction between nicotine and the mesenchymal and oral ectoderm during odontogenesis can disrupt the normal process of tooth development and lead to delays. Toxins like nicotine can also cause disturbances in dental germ development, which is highly sensitive to environmental factors. Maternal consumption of substances like morphine can further exacerbate abnormal tooth development. Our study found that newborns from the experimental group had significantly lower papillary thickness compared to the control group, possibly due to exposure to insecticides, nicotine, and morphine. Our findings support the hypothesis that morphine consumption can reduce the thickness of enamel matrix and dentin, although further investigation is needed to determine the exact mechanism of its effect.

 The long stages of project approval, limitations in funding, and morphine supply were among the limitations that were minimized with the consultation and assistance of Kerman University’s esteemed Vice Chancellor for Research.

## Conclusion

 Based on our analysis of dental germ evolution, the experimental group demonstrated a delay in the amelogenesis and dentinogenesis stages compared to the control group. Consequently, the enamel and dentin thickness formed in the experimental group was lower than in the control group. The experimental group underwent significant dental germ development involving morphological and tissue differentiation processes. The dental germ in the experimental group had all the essential components required for the future crown of teeth, along with differentiated tissues capable of secreting hard tissues such as enamel and dentin.

## Competing Interests

 The authors of this manuscript certify that they have no conflicts of interest.

## Ethical Approval

 This study was approved by the Ethics Committee of Kerman University of Medical Sciences under the code IR.KMU.REC.1398.488.

## References

[1] Badshah I, Anwar M, Murtaza B, Khan MI (2024). Molecular mechanisms of morphine tolerance and dependence; novel insights and future perspectives. Mol Cell Biochem.

[2] Plein LM, Rittner HL (2018). Opioids and the immune system - friend or foe. Br J Pharmacol.

[3] World Health Organization (WHO). Information Sheet on Opioid Overdose. 2019. Available from: https://www.who.int/substance_abuse/information-sheet/en/. Accessed August 22, 2019.

[4] Shams Lahijani M, Ahmadzadeh F, Dabiri M (2006). Teratogenic effects of a new quinazolinone derivative on the development of BALB/c mice fetuses on days 9 10 and 11 of gestation. Iran J Sci Technol Trans A Sci.

[5] Bosworth OM, Padilla-Azain MC, Adgent MA, Spieker AJ, Wiese AD, Pham A (2024). Prescription opioid exposure during pregnancy and risk of spontaneous preterm delivery. JAMA Netw Open.

[6] Berríos-Cárcamo P, Quezada M, Santapau D, Morales P, Olivares B, Ponce C (2022). A novel morphine drinking model of opioid dependence in rats. Int J Mol Sci.

[7] Saeidinezhad M, Razban V, Safizadeh H, Ezzatabadipour M (2021). Effects of maternal consumption of morphine on rat skeletal system development. BMC Musculoskelet Disord.

[8] Butler PM (1967). Dental merism and tooth development. J Dent Res.

[9] Sant’Anna LB, de Oliveira Tosello D (2017). A histomorphometrical study of the effects of ethanol on enamel formation in rat mandibular molars during pregnancy. Braz J Morphol Sci.

[10] Chowdhury IG, Bromage TG (2000). Effects of fetal exposure to nicotine on dental development of the laboratory rat. Anat Rec.

[11] Silva IH, Leão JC, Evêncio LB, Porter SR, de Castro RM (2010). Morphological analysis of the enamel organ in rats treated with fluoxetine. Clinics (Sao Paulo).

[12] Avsar A, Topaloglu B, Hazar-Bodrumlu E (2013). Association of passive smoking with dental development in young children. Eur J Paediatr Dent.

[13] Kameli S, Moradi-Kor N, Tafaroji R, Ghorbani R, Farzadmnesh H, Sameni H (2019). Effects of amoxicillin on the structure and mineralization of dental enamel and dentin in Wistar rats. Front Dent.

[14] Abbassy MA, Watari I, Bakry AS, Hamba H, Hassan AH, Tagami J (2015). Diabetes detrimental effects on enamel and dentine formation. J Dent.

[15] de Souza JF, Gramasco M, Jeremias F, Santos-Pinto L, Giovanini AF, Cerri PS (2016). Amoxicillin diminishes the thickness of the enamel matrix that is deposited during the secretory stage in rats. Int J Paediatr Dent.

[16] Sant’Anna LB, Tosello DO, Pasetto S (2005). Effects of maternal ethanol intake on immunoexpression of epidermal growth factor in developing rat mandibular molar. Arch Oral Biol.

[17] Li X, Sundquist J, Kane K, Jin Q, Sundquist K (2010). Parental occupation and preterm births: a nationwide epidemiological study in Sweden. Paediatr Perinat Epidemiol.

[18] Hernández Guerrero JC, Portilla Robertson J, Ledezma Montes C, Ponce-Bravo S, Miranda Gómez A, Arias Rivera EM (1996). Immunoexpression of epidermal growth factor in odontogenesis of the offspring of alcoholic mice. Bol Estud Med Biol.

[19] Campos RM (1998). Effects of prolonged ethanol use on the early stages of dental development in mice. Rev Cuba Invest Biomed.

[20] Aycicek A, Erel O, Kocyigit A (2005). Increased oxidative stress in infants exposed to passive smoking. Eur J Pediatr.

[21] Abe T, Miyajima H, Okada K (2003). Effects of a macrolide antibiotic on enamel formation in rat incisors--primary lesion of ameloblast at the transition stage. J Vet Med Sci.

[22] Westergaard J (1980). Structural changes induced by tetracycline in secretory ameloblasts in young rats. Scand J Dent Res.

[23] Bronckers AL, Lyaruu DM, DenBesten PK (2009). The impact of fluoride on ameloblasts and the mechanisms of enamel fluorosis. J Dent Res.

[24] Kuscu OO, Sandalli N, Dikmen S, Ersoy O, Tatar I, Turkmen I (2013). Association of amoxicillin use and molar incisor hypomineralization in piglets: visual and mineral density evaluation. Arch Oral Biol.

[25] Jeremias F, Koruyucu M, Küchler EC, Bayram M, Tuna EB, Deeley K (2013). Genes expressed in dental enamel development are associated with molar-incisor hypomineralization. Arch Oral Biol.

[26] Vardavas CI, Chatzi L, Patelarou E, Plana E, Sarri K, Kafatos A (2010). Smoking and smoking cessation during early pregnancy and its effect on adverse pregnancy outcomes and fetal growth. Eur J Pediatr.

[27] Hernandez-Guerrero JC, Ledesma-Montes C, Loyola-Rodriguez JP (1998). Effects of maternal ethanol intake on second alcoholic generation murine skull and mandibular size. Arch Med Res.

